# How Do Education and Family Planning Accelerate Fertility Decline?

**DOI:** 10.1111/padr.12347

**Published:** 2020-07-23

**Authors:** Daphne H. Liu, Adrian E. Raftery

## Abstract

Education and family planning can both be influenced by policy and are thought to accelerate fertility decline. However, questions remain about the nature of these effects. Does the effect of education operate through increasing educational attainment of women or educational enrollment of children? At which educational level is the effect strongest? Does the effect of family planning operate through increasing contraceptive prevalence or reducing unmet need? Is education or family planning more important?

We assessed the quantitative impact of education and family planning in high‐fertility settings using a regression framework inspired by Granger causality. We found that women's attainment of lower secondary education is key to accelerating fertility decline and found an accelerating effect of contraceptive prevalence for modern methods. We found the impact of contraceptive prevalence to be substantially larger than that of education. These accelerating effects hold in sub‐Saharan Africa, but with smaller effect sizes there than elsewhere.

## Introduction

The United Nations projects that world population will increase from its present 7.7 billion to 10.9 billion people in 2100, with more than half of this increase in sub‐Saharan Africa (SSA), mostly in high‐fertility countries (United Nations 2019c). Much of the rest of the increase will be in countries in Asia and Latin America with above‐replacement fertility. It is widely thought that these countries would benefit from a slower population increase brought about by a more rapid decrease in fertility, as high fertility and rapid population growth are likely to have adverse economic, environmental, health, governmental, and political consequences (Bongaarts 2013). Declining fertility can also yield a demographic dividend by reducing the dependency ratio, increasing women's participation in the paid labor force, and allowing increased investments in human and physical capital (Lee and Mason 2006; Mason and Lee 2006). This raises the question of how the fertility decline could be accelerated in high‐fertility countries. There is widespread agreement in the literature that there are two main factors that can be influenced by policy and may help accelerate fertility decline: education and family planning (Hirschman 1994).

Increased education is thought to accelerate fertility decline through two main mechanisms (Hirschman 1994; Axinn and Barber 2001). The first is by increasing the opportunity cost for women, measured by their educational attainment, of having children. Demand or structural theories of fertility decline, such as Easterlin and Crimmins (1985), argue that educated women have higher status and access to opportunities, thus increasing the opportunity costs of childbearing. Ideational theories argue that increased parental schooling changes the value placed on large family size and spreads information about family planning, increases consumption aspirations, and spreads Western family values (Axinn and Barber 2001). Many studies of the relationship between education and fertility, particularly on the causal nature of this relationship, focus on the mechanism of women's educational attainment (e.g., Martin 1995; Raftery, Lewis, and Aghajanian 1995; Osili and Long 2008; Cygan‐Rehm and Maeder 2013; Behrman 2015; Bongaarts, Mensch, and Blanc 2017). Women's attainment is the mechanism proposed by Lutz, Butz, and Samir (2014) in their argument for the importance of including education in population projections.

Education may also accelerate fertility decline by increasing the cost of raising children, which can be measured via the enrollment rates of children. The intergenerational wealth flows theory (Caldwell 1982; Caldwell, Reddy, and Caldwell 1985) argues for the importance of children's education, stating that children's schooling reduces their capacity for work and increases the cost of childrearing. Microeconomic theories based on the “quantity‐quality tradeoff” have also emphasized the role of children's enrollment in shaping parents’ future childbearing decisions (Axinn and Barber 2001). Easterlin and Crimmins (1985) also acknowledge the role the cost of raising children may play in parents’ future childbearing decisions. Studies that evaluate the impact of children's enrollment on fertility decline include Raftery et al. (1995), Subbarao and Raney (1995), and Masih and Masih (2000).

Family planning is the second quantity that can be influenced by policy and may accelerate fertility decline. Although education and other factors may change fertility preferences, family planning is needed to translate those changed preferences into changes in fertility. As one of the most important proximate determinants of fertility (Bongaarts 1987), contraceptive use can provide a means by which individuals can attain their desired childbearing. Studies have consistently found a strong negative association between contraceptive prevalence and fertility (Tsui 2001; Bongaarts 2010).

While the potential mechanisms by which increased education or family planning may affect fertility decline are well known, there is less consensus on the relative impact the different mechanisms may have. There is a well‐documented positive association between education and family planning (Ainsworth, Beegle, and Nyamete 1996; Kirk and Pillet 1998; Bongaarts 2010), where more educated women have a higher demand for and greater use of family planning. Using demographic and health surveys (DHS) data, Martin (1995) found that differentials in contraceptive use between education groups tended to be smaller when the overall contraceptive prevalence in a country was higher. Masih and Masih (2000) found that both the female secondary gross enrollment ratio and contraceptive use as measured by female sterilization had a significant impact on fertility in India over the period 1965–1991, where the combined effect of education and family planning explained, in a Granger‐causal sense, a substantial portion of the variability in the total fertility rate (TFR). However, Masih and Masih found that only education was exogenous. Through simulations and using data from the 1993 Indonesia Family Life Survey, Angeles, Guilkey, and Mroz (2005) found a larger effect of family planning programs on reducing fertility than improvements in school quality.

An additional consideration when evaluating the relative effects of education and family planning on fertility decline is that contraception is a proximate determinant of fertility while education is not. Women's education has been hypothesized to have an indirect effect on fertility decline through family planning, as increased education may affect fertility by increasing knowledge of family planning (Cochrane 1979) or by changing attitudes towards its acceptability (Cleland and Wilson 1987). Evidence of this indirect effect has been seen in Indonesia, where Gertler and Molyneaux (1994) found much of the effect of increased women's educational attainment on fertility decline in the 1980s was an indirect effect through contraceptive use.

**FIGURE 1:**
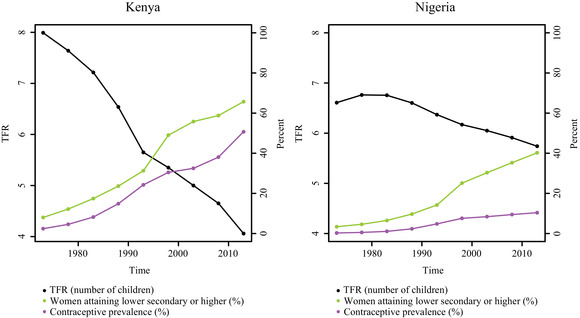
TFR (black), percentage of women who have attained at least lower secondary education or higher (green), and contraceptive prevalence (purple) for Kenya from 1970–1975 to 2010–2015 and Nigeria from 1975–1980 to 2010–2015

Figure 1 shows trends in the TFR, the percentage of women who have attained lower secondary education or higher, and the prevalence of modern contraceptive methods for Kenya and Nigeria from 1975–1980 to 2010–2015. The decline in TFR from 1970 onwards has been faster in Kenya than in Nigeria. Correspondingly, we see faster growth in women's educational attainment and contraceptive prevalence in Kenya than in Nigeria. Notably, educational attainment and contraceptive prevalence have followed similar growth trajectories in Kenya while educational attainment has grown at a faster rate than contraceptive prevalence in Nigeria.

We are interested in whether the effect of education operates primarily through increased educational attainment of women or through increased educational enrollment of children. Identifying at which educational level the impact is strongest is also of interest, especially from a policy standpoint. We also aim to evaluate whether the impact of family planning on fertility decline operates by reducing unmet need for family planning or by increasing contraceptive prevalence. This distinction, while subtle, is vital for crafting effective family planning policies.

Finally, we are interested in quantifying the relative impacts of education and family planning on fertility decline. We will evaluate if increasing education or family planning accelerates fertility within a high‐fertility context; that is, whether increases in education or family planning correspond to declines in TFR faster than what we would already expect given historical trends. If so, we aim to identify which education mechanism, level of education, and measure of family planning has the strongest effects.

We also explore the accelerating effect of covariates on fertility decline within SSA compared to other regions of the world. The SSA fertility transition has been slower than historical fertility transitions in Asia and Latin America, and fertility decline has even stalled in many parts of SSA (Bongaarts and Casterline 2013). Countries in SSA may be experiencing different relationships between education, family planning, and fertility compared to other historically high‐fertility regions. There is evidence of differences in ideal family size, which may diminish the effect of family planning in SSA (Bongaarts, Frank, and Lesthaeghe 1984; Bongaarts and Casterline 2013). There also appear to be differences in school quality, which may diminish the effect of education in SSA (Grant 2015). We do not explicitly use measures of ideal family size or school quality, but these hypotheses for the SSA difference motivate our work.

## Data

We use estimates of TFR from the United Nations World Population Prospects (WPP) 2019 Revision (United Nations 2019c), which is available for 201 countries by five‐year time periods. As we are interested in estimating relationships spanning all current and historical high‐fertility transitions, we need estimates of TFR that are comparable across countries and time periods. As recommended by Bongaarts (2017), we use estimates of TFR from the United Nations. The estimates of TFR from WPP are based on vital registers, censuses, and surveys such as the DHS and the multi‐indicator cluster surveys. These individual data sources are available on an uneven basis across countries and across time and often have known biases or data quality issues that need to be adjusted for. For example, DHS fertility estimates suffer from inconsistent data quality across countries, often due to misreporting or omission of recent births (Schoumaker 2014). The WPP estimates account for these adjustments and allow us to use information drawing from multiple data sources.

We denote the TFR for country *c* in five‐year time period *t* by $f_{c,t}$. Decrements in TFR are constructed as a measure of fertility decline, with the TFR decrement from five‐year time period (t−1) to five‐year time period *t* defined as $\Delta f_{c,t}=f_{c,t-1}-f_{c,t}$. This assigns larger positive values to the TFR decrement when fertility is declining faster. As we are mainly interested in changes to the rate of fertility decline, we focus on modeling the TFR decrement. Our outcome variable is thus on the timescale of differences in five‐year time periods. Correspondingly, we expect any covariates we add to the model to be on the scale of changes over time.

We construct these changes so they are positive when the covariate is “improving.” For example, if an education covariate *X* is increasing over time on average, we define the corresponding change as $\Delta X_{c,t}=X_{c,t}-X_{c,t-1}$. This ensures that $\Delta X_{c,t}$ is positive when education is increasing in country *c*. Changes over time in education, family planning, urbanization, and GDP variables were defined analogously to $\Delta X_{c,t}$. The change over time in child mortality (_5_ *q*_0_) was defined to be in the same direction as the TFR decrement. For country *c* and five‐year time periods (t−1) and *t*, the decrement in child mortality was constructed as ${\left({}_{5}q_{0}\right)}_{c,t-1}-\;{\left({}_{5}q_{0}\right)}_{c,t}$.

We identify a “high‐fertility transition” subset of our data to serve as the main focus of our analyses. For each country, we are primarily interested in data corresponding to time periods where the country was in Phase II of the fertility transition as defined by Alkema et al. (2011) and had a TFR greater than 2.5. This results in a subset of 666 country‐time period pairs with observations from 121 countries. The earliest time period for which we have data is 1970–1975.

We consider two measures of education: women's educational attainment and children's enrollment. Educational attainment data for women aged 20–39 were obtained from the Wittgenstein Centre (2018). The Wittgenstein Centre provides a harmonized dataset of the educational attainment distribution using six levels of attainment: no education, incomplete primary, primary, lower secondary, upper secondary, and postsecondary. These attainment levels are constructed to be comparable across countries and times and are based on the International Standard Classification of Education. We focus on cumulative levels of attainment such as the proportion of women who completed primary education or higher. Data on children's enrollment are obtained from the World Bank (2019). Net enrollment rates (NER) for primary and secondary education for both sexes combined are available from 1970 onwards. Missing NER values are imputed using a combination of a piecewise LOESS curve based on the gross enrollment ratio, also from the World Bank, and linear interpolation.

We consider the median estimates of contraceptive prevalence and unmet need for family planning from the United Nations Estimates and Projections of Family Planning Indicators 2019 (United Nations 2019a), based on the methodology of Alkema et al. (2013). These family planning indicators are available for married or in‐union women aged 15–49 years beginning from 1970. Contraceptive prevalence and unmet need are reported as percentages of the total number of married or in‐union women. We convert these percentages to proportions between 0 and 1 for analyses.

Finally, we consider several control variables. Estimates of child mortality (_5_ *q*_0_) are obtained from WPP 2019, where we exclude mortality data from the time periods corresponding to the genocides in Cambodia and Rwanda. We also consider measures of GDP per capita growth (as percent growth) from the World Bank and the percentage of population residing in urban areas from the UN World Urbanization Prospects 2018 (United Nations 2018). GDP and urbanization measures are converted to proportions between 0 and 1 for analyses.

**FIGURE 2:**
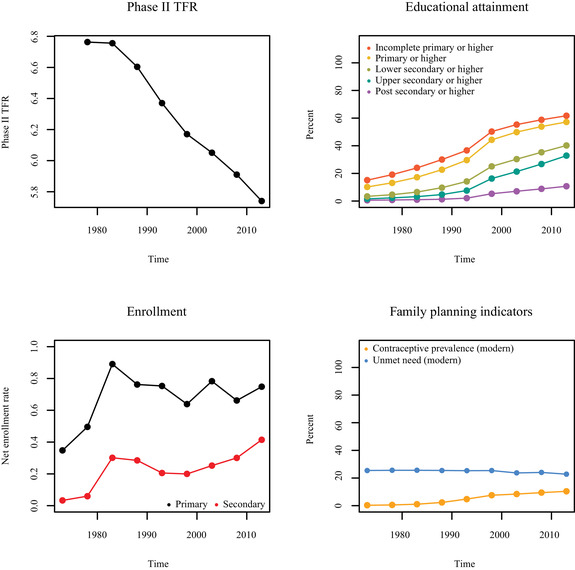
Trends in Phase II TFR, cumulative educational attainment, NER, and family planning indicators for modern methods in Nigeria from 1975–1980 to 2010–2015

**FIGURE 3:**
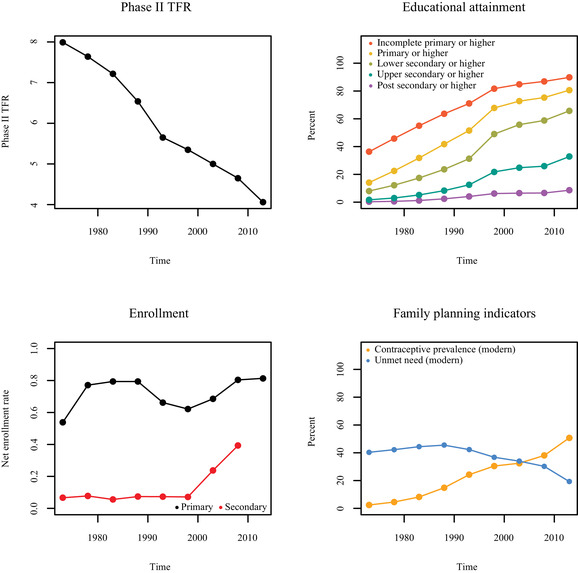
Trends in Phase II TFR, cumulative educational attainment, NER, and family planning indicators for modern methods in Kenya from 1975–1980 to 2010–2015

Examples of trends in TFR, women's attainment, children's enrollment, and family planning indicators for Nigeria and Kenya can be seen in Figures 2 and 3, respectively. We see that Nigeria has experienced a slow but steady decrease in TFR over time. Increases in women's educational attainment have mostly occurred from the 1990s onwards, though increased enrollment rates are seen early on. Nigeria has experienced relatively small improvements in family planning indicators for modern contraceptive methods since 1970. Kenya is an example of a sub‐Saharan African country that has experienced a more rapid fertility decline than Nigeria. There have also been larger increases in women's educational attainment, particularly of lower secondary education, and larger increases in access to modern methods of family planning in Kenya than in Nigeria. However, the increase in enrollment rates in secondary education has been notably delayed in Kenya.

## Methodology

### Modeling framework

Our methodology draws inspiration from Granger causality to answer questions about how covariates may affect the acceleration of fertility decline. Granger causality is based on the assumption that the cause must temporally precede the effect. A covariate *X* is said to “Granger‐cause” the outcome *Y* if *X* can provide additional information for forecasting *Y* that is not already captured in past values of *Y* (Granger 1969). Following this logic, to investigate if education or family planning covariates have an accelerating effect on fertility decline beyond what we would already expect the decline to look like based on past trends, we need to include a measure of the “expected fertility decline” in our model. This measure of expected decline should be based on past TFR trends and be both country‐ and time‐specific. We draw from the Bayesian hierarchical model that is the basis of the model for probabilistic fertility projections currently used by the UN (United Nations, 2017; Alkema et al. 2011; Raftery, Alkema, and Gerland 2014; Fosdick and Raftery 2014).

In the Bayesian hierarchical model, the expected TFR decrement from five‐year time period (t−1) to five‐year time period *t* is modeled as a double logistic function. For country *c* and time period *t*, the double logistic function is defined as

$$\begin{array}{ccc} g\left(\theta_{c},f_{c,t}\right) & = & \frac{-d_{c}}{1\;+\;\text{exp}\left(-\frac{2\ln\left(9\right)}{\Delta_{c,1}}\left(f_{c,t}-\Sigma_{i\;=\;1}^{4}\Delta_{c,i}\;+\;0.5\Delta_{c,1}\right)\right)}\\ & & +\;\frac{d_{c}}{1\;+\;\text{exp}\left(-\frac{2\ln\left(9\right)}{\Delta_{c,3}}\left(f_{c,t}-\Delta_{c,4}-0.5\Delta_{c,3}\right)\right)}. \end{array}$$

In the double logistic function, $\theta_{c}=\;\left(\Delta_{c,1},\Delta_{c,2},\Delta_{c,3},\Delta_{c,4},d_{c}\right)$ is a vector of country‐specific parameters. For country *c*, the parameter $d_{c}$ represents the maximum possible five‐year TFR decrement. The parameters $\Delta_{c,i}$ for i=1,⋯,4 describe the range of TFR values in which the pace of the fertility decline changes. Specifically, the start of the fertility transition occurs at TFR level $U_{c}=\Sigma_{i=1}^{4}\;\Delta_{c,i}$. At this TFR, the pace of the decline is around $0.1d_{c}$. From TFR levels $U_{c}$ to $U_{c}-\Delta_{c,1}$, the pace of the fertility decline increases to at least $0.8d_{c}$. The pace of fertility decline is the highest for the TFR values denoted by $\Delta_{c,2}$, where it ranges from $0.8d_{c}$ to $d_{c}$. During the TFR range $\Delta_{c,3}$, the pace of fertility decline decreases and by TFR level $\Delta_{c,4}$ the pace of decline has decreased to $0.1d_{c}$.

The expected TFR decrement is incorporated into the Bayesian hierarchical model

$$\begin{array}{ccc} f_{c,t} & = & f_{c,t-1}-g\left(f_{c,t-1}|\theta_{c}\right)+\varepsilon_{c,t}\\ \varepsilon_{c,t} & \overset{\mathrm{iid}}{∼} & N\left(0,\sigma {\left(f_{c,t-1}\right)}^{2}\right)\\ \theta_{c}\; & ∼ & h\left(\cdot ,\phi \right)\\ \phi & ∼ & \pi \left(\cdot \right), \end{array}$$

where the country‐specific parameter vector $\theta_{c}$ follows a world distribution h(·|ϕ) with parameter ϕ. The prior distribution of ϕ is π(·). We used the median of the posterior distribution of this double logistic function from the Bayesian hierarchical model as our “expected fertility decline” covariate.

### Modeling correlation

There is between‐country correlation in TFR that must be accounted for in our model (Fosdick and Raftery 2014). However, estimating correlation matrices directly can lead to noisy estimates since we have 121 countries and each country has at most eight observations. Thus, we cannot simply use the empirical correlation estimates.

**FIGURE 4:**
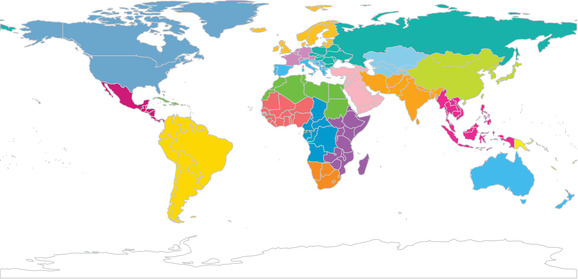
The 22 UN regions used for the GLS clustering scheme

We modeled the between‐country correlation based on UN region membership. The UN regions are displayed in Figure 4. Each UN region is a set of countries that are relatively close geographically and homogeneous culturally. We expect there to be similar between‐country correlation for all countries in the same UN region and at the same time point. We used generalized least squares (GLS) to fit our models via maximum likelihood, as GLS allows us to introduce a between‐country correlation structure by constructing clusters based on UN region × time point combinations from the 22 UN regions and eight time points. We assumed an exchangeable correlation structure within each UN region × time point cluster. This correlation structure implies that countries within the same UN region have the same amount of between‐country correlation at a given time point. We also assumed homoscedastic errors and between‐cluster independence.

Our model is specified as follows. Let $f_{r,t}$ represent the vector of TFR values for all countries in UN region *r* at time *t*, let $g_{r,t}$ represent the vector of the expected TFR decrement for all countries in region *r* at time *t*, and let $H_{r,t}$ represent the matrix of covariates for all countries in region *r* at time *t*. Our model can then be written as

(1) $$\begin{array}{ccc} \Delta f_{r,t}\; & = & f_{r,t-1}-f_{r,t}\\ & = & \;\beta_{0}+\beta_{1}g_{r,t}+\beta_{h}H_{r,t}+\varepsilon_{r,t}\\ \varepsilon_{r,t} & ∼ & N\left(0,\Sigma_{r,t}\right) \end{array},$$

where the (i,j)th term of $\Sigma_{r,t}$ represents the covariance between countries *i* and *j* from region *r* at time *t*. We model $\Sigma_{r,t}$ as

$$\Sigma_{r,t}\;=\sigma^{2}R_{r,t},\;\mathrm{where}\;R_{r,t}=\left\{\begin{array}{cc} 1 & \mathrm{if}\;i=j\\ \rho & \mathrm{if}\;i\neq j \end{array}.\right.$$

### Model selection

We determined which measures of education and family planning to include in our model for TFR decrement using the Bayesian information criterion (BIC) as the model selection criterion (Schwarz 1978; Raftery 1995). We aimed to answer three main questions with the model selection. First, does education affect fertility decline primarily through increased educational attainment of women or through increased enrollment of children? Second, given the selected education mechanism, which levels of education are the most important? Third, given the selected mechanism and levels of education, does family planning affect fertility decline primarily through reducing unmet need for family planning or though increasing contraceptive prevalence? Using a BIC‐based model selection process, we identified which measures of education and family planning were favored by the data.

The model selection process selected one education variable, namely the change over time in women's completion of lower secondary education or higher. This implies that the driving mechanism behind the education effect on fertility is the mother's education rather than children's enrollment. It also implies that the key levels of education are completion of lower secondary education or higher.

The model selection process also selected one family planning variable, namely the change over time in prevalence of modern contraceptive methods. This suggests that contraceptive prevalence captures the driving mechanism behind the family planning effect better than unmet need for family planning.

We did not use the BIC to determine which control variables to include in our model. Instead, the control variables measuring child mortality, urbanization, and GDP were included as important background variables to consider, based on evidence in the literature. We also included an indicator variable for SSA:

$$\begin{array}{cc} \mathrm{SS}A_{c,t}= & \left\{\begin{array}{cc} 1 & \mathrm{if}\;\mathrm{country}\;c\;\mathrm{is}\;\mathrm{in}\;\mathrm{SSA}\\ 0 & \mathrm{if}\;\mathrm{country}\;c\;\mathrm{is}\;\mathrm{not}\;\mathrm{in}\;\mathrm{SSA}. \end{array}\right. \end{array}\;$$

The selected variables and their abbreviated names can be found in Table 1. For the BIC‐based model selection, all models were fitted using GLS, following Equation (1). All models included the SSA indicator, Expected TFR Decr, GDP Growth, GDP Growth Change, Urban Change, and Child Mortality Decr as covariates. All continuous variables were centered prior to model fitting.

**TABLE 1:** Abbreviated names and descriptions of BIC‐selected measures of education and family planning and all control variables

Name	Description
Expected TFR Decr	Expected TFR decrement from Alkema et al. (2011)
LowSec+ Change	Change over time in proportion of women who have attained lower secondary education or higher
CP (Modern) Change	Change over time in contraceptive prevalence of modern methods
SSA	Indicator for whether a country is in sub‐Saharan Africa
GDP Growth	Annual percentage growth rate of GDP per capita at market prices based on constant local currency
GDP Growth Change	Change over time of GDP Growth
Urban Change	Change over time of the percent of population residing in urban areas
Child Mortality Decr	Change over time of under‐five mortality (_5_ *q*_0_)

#### Model selection for education

The education variables considered in the model selection were the change over time in NER and the cumulative levels of change over time in women's educational attainment. We only considered cumulative levels of women's attainment since changes in noncumulative levels are difficult to interpret in terms of overall educational gains. For example, an increase in the proportion of women who have attained at most lower secondary education could correspond to more women moving from completing only primary to completing lower secondary education, which would indicate an overall improvement in women's education. However, this increase could also correspond to fewer women moving from lower secondary to upper secondary education, which would indicate an overall decrease in women's education. Using cumulative levels of change over time in attainment eliminates this interpretation problem, as an increase in the proportion of women who have attained lower secondary education or higher unambiguously indicates an overall improvement in women's education.

We first consider the selection of women's attainment over children's enrollment. Due to the limited availability of enrollment data, models including enrollment are based on a reduced dataset of 550 country‐time pairs with observations from 116 countries. We selected one education variable, the change over time in the proportion of women who have attained lower secondary education or higher (“LowSec+ Change”), from among the six levels of the change over time in cumulative levels of women's attainment (incomplete primary or higher, primary or higher, lower secondary or higher, upper secondary or higher, and postsecondary) and both levels of the change over time of NER (primary and secondary) using BIC as the model selection criterion. The first column of Table 2 summarizes the model including both women's attainment and children's enrollment variables. The different levels of women's attainment are abbreviated analogously to LowSec+ for lower secondary or higher. The change over time in NER is abbreviated to NER Change.

**TABLE 2:** Education variable selection: summaries of the model with all education variables and the model with only attainment variables, where both models include all control variables and are fit by GLS with TFR decrement as the dependent variable

	Model with attainment and enrollment		Model with attainment only	
	Estimate	*t*‐value	Estimate	*t*‐value
(Intercept)	0.33	17.4^***^	0.30	16.3^***^
Expected TFR Decr	0.91	18.4^***^	0.91	19.2^***^
IncPri+ Change	−0.66	−1.3	−0.73	−1.5
Pri+ Change	0.79	1.3	1.00	1.8
LowSec+ Change	2.11	3.2^**^	2.38	3.9^***^
UppSec+ Change	−0.78	−1.0	−1.04	−1.5
PostSec+ Change	0.63	0.5	−0.49	−0.5
NER Change (Pri)	−0.16	−1.1		
NER Change (Sec)	−0.16	−1.6		
GDP Growth	−0.38	−1.4	−0.45	−1.7^*^
GDP Growth Change	0.55	2.9^**^	0.46	2.6^*^
Urban Change	−0.31	−0.6	−0.47	−1.0
Child Mortality Decr	0.07	0.1	0.70	0.9
SSA	−0.08	−2.5^*^	−0.09	−2.6^**^
Within‐cluster correlation	0.23	0.30		
*R* ^2^	0.52		0.49	
BIC	−52.30		−86.45	
Country‐time pairs	550		666	

^***^denotes *P* < 0.001, ^**^denotes *P* < 0.01, and ^*^denotes *P* < 0.05.

In the model including NER Change, we found the only significant education variable was LowSec+ Change. Neither of the variables measuring children's enrollment was significant. Since we constructed the “change over time” education variables to be positive when education is increasing, we expected to find positive coefficient estimates for the education variables. However, we found that several of the coefficient estimates, including the coefficient estimates for both enrollment variables, were negative.

From these results, we have answered our first question of interest and found that women's attainment was selected over children's enrollment. We have also answered our second question, as the only significant levels of attainment corresponded to the levels lower secondary or higher. However, due to the limited availability of data on children's enrollment, the model including NER Change was fitted using only 550 country‐time pairs. Given that the selected education mechanism was attainment, we confirmed that the selected levels of attainment were truly lower secondary or higher once we considered all 666 country‐time pairs. The second column of Table 2 summarizes the model with all levels of women's attainment but not including children's enrollment. We once again found LowSec+ Change was the only significant education variable, supporting our choice of LowSec+ Change as the selected education variable.

Although we selected from the cumulative parameterization of the change over time in women's attainment, we additionally verified the selection of LowSec+ Change by checking the estimated effects for the noncumulative parameterization. Details of this verification can be found in the online supplementary Appendix.

#### Model selection for family planning

Finally, we consider the third model selection question: Given that LowSec+ Change is the selected education variable, does family planning affect fertility decline primarily by reducing unmet need for family planning or by increasing contraceptive prevalence? We considered only contraceptive prevalence and unmet need in the selection of family planning indicators despite the availability of a third indicator, demand for family planning satisfied, from the UN. Estimates of contraceptive prevalence and unmet need are both available as percentages of the total number of married or in‐union women. Estimates of demand for family planning satisfied are available as a percentage of the total number of married or in‐union women who are using any method of contraception or are having an unmet need for family planning (United Nations 2019b). This difference in denominator is not ideal for direct comparisons of demand for family planning satisfied with the other family planning indicators. All three UN family planning indicators suffer from the “exposure to risk of pregnancy” limitation as argued by Bongaarts (2017), as the family planning indicators are measured among married or in‐union women, while the TFR measures births among all women. Demand for family planning satisfied suffers an additional limitation in this regard since it further restricts the group of women considered. For these reasons, we considered only contraceptive prevalence and unmet need as our potential family planning indicators.

The UN provides estimates of contraceptive prevalence for all methods, modern methods, and traditional methods, and estimates of unmet need for all methods and modern methods. These different estimates are highly correlated. Using BIC, we selected the change over time in contraceptive prevalence of modern methods, denoted CP (Modern) Change, from among the five family planning indicators. The estimates of contraceptive prevalence from the UN do not consider contraceptive effectiveness, which Bongaarts (2017) identifies as a key limitation in analyzing the relationship between contraceptive prevalence and TFR. The selection of contraceptive prevalence of modern contraceptive methods partially addresses the issue of differential contraceptive effectiveness, as the least effective methods (traditional methods) are omitted.

We compared our selected family planning indicator of CP (Modern) Change with the change over time in unmet need for family planning, which is the measure of family planning discussed by Bongaarts and Casterline (2013). For comparison purposes, we considered only the change over time in unmet need for modern methods, here called Unmet Need (Modern) Change. Table 3 summarizes the model including both CP (Modern) Change and Unmet Need (Modern) Change. We found that the effect of Unmet Need (Modern) Change was not significant and that its effect size was smaller than that of CP (Modern) Change, supporting our selection of family planning indicator.

**TABLE 3:** Family planning variable selection: summary of model with contraceptive prevalence, unmet need for family planning, the BIC‐selected education variable, and all control variables, fit by GLS with TFR decrement as the dependent variable

	Estimate	*t*‐value
(Intercept)	0.31	19.4^***^
Expected TFR Decr	0.82	18.1^***^
LowSec+ Change	1.53	4.7^***^
CP (Modern) Change	2.74	7.4^***^
Unmet Need (Modern) Change	0.15	0.3
GDP Growth	−0.58	−2.4^*^
GDP Growth Change	0.51	3.0^**^
Urban Change	−0.35	−0.8
Child Mortality Decr	−0.17	−0.2
SSA	−0.07	−2.4^*^
Within‐cluster correlation	0.25	
*R* ^2^	0.55	
BIC	−180.43	
Country‐time pairs	666	

^***^denotes *P* < 0.001, ^**^denotes *P* < 0.01, and ^*^denotes *P* < 0.05.

## Results

We first fit the model in Equation (1) with main effects only via GLS for the BIC‐selected education and family planning variables and all control variables. Next, to identify the potentially differential effect of the covariates on fertility decline within SSA compared to the rest of the world, we considered interaction terms between the SSA indicator and the BIC‐selected education and family planning variables and all control variables. We did not consider an interaction between SSA and Expected TFR Decr because the expected decrement is already country‐specific by construction. The model with all interactions with SSA is summarized in the first column of Table 4, and the model with main effects only is summarized in the second column of Table 4. All continuous variables were centered prior to fitting these models.

**TABLE 4:** Final models with BIC‐selected education and family planning covariates, all control variables, and with and without interactions with the SSA indicator, fit by GLS with TFR decrement as the dependent variable

	Including interactions with SSA		Main effects only	
	Estimate	*t*‐value	Estimate	*t*‐value
(Intercept)	0.31	19.6^***^	0.31	19.5^***^
Expected TFR Decr	0.81	18.3^***^	0.82	19.1^***^
LowSec+ Change	1.79	4.6^***^	1.52	4.8^***^
CP (Modern) Change	3.38	9.3^***^	2.67	9.8^***^
GDP Growth	−1.20	−3.3^**^	−0.58	−2.4^*^
GDP Growth Change	0.77	3.7^***^	0.51	3.0^**^
Urban Change	−1.46	−2.5^*^	−0.35	−0.8
Child Mortality Decr	0.44	0.4	−0.14	−0.2
SSA	−0.06	−2.1^*^	−0.07	−2.4^*^
SSA:LowSec_ Change	−0.57	−0.8		
SSA:CP (Modern) Change	−1.55	−2.8^**^		
SSA:GDP Growth	1.08	2.1^*^		
SSA:GDP Growth Change	−0.61	−1.7		
SSA:Urban Change	1.81	2.1^*^		
SSA:Child Mortality Decr	−1.40	−0.9		
Within‐cluster correlation	0.23		0.25	
*R* ^2^	0.57		0.55	
BIC	−168.49		−186.85	
Country‐time pairs	666		666	

^***^denotes P<0.001, ^**^denotes P<0.01, and ^*^denotes P<0.05.

In both models, we found a significant positive relationship between TFR decrement and LowSec+ Change, where larger increases in the proportion of women who have attained lower secondary education or higher were associated with larger decrements in TFR and thus faster fertility decline. In other words, we found an accelerating effect of women's attainment of lower secondary or higher education on fertility decline. Similarly, we found an accelerating effect of contraceptive prevalence of modern methods on fertility decline.

We found separate significant effects of women's educational attainment and contraceptive prevalence even after accounting for the expected TFR decrement and control variables. This follows our expectations from the literature, where generally it has been found there are significant independent effects of family planning and socioeconomic conditions like that of education on fertility (Hirschman 1994).

Although we found that education and family planning are both important for accelerating fertility decline beyond what we already expect based on past trends, the magnitudes of the coefficient estimates indicate that faster increases in contraceptive prevalence were associated with larger gains in the rate of fertility decline than faster increases in educational attainment. In the model with interactions, the effect of a change in CP (Modern) Change on TFR decrement was slightly less than twice what an equivalent change in LowSec+ Change would have on TFR decrement. Note that the regression coefficients for LowSec+ Change and CP (Modern) Change can be compared because they are both on the same scale.

We found the larger effect size for contraceptive prevalence compared to educational attainment still held when we considered the composite effects of the observed values and coefficient estimates of CP (Modern) Change and LowSec+ Change. In our dataset, there has been more rapid observed change in educational attainment than in contraceptive prevalence. The median observed value of LowSec+ Change was 0.045, while the median observed value of CP (Modern) Change was 0.034. As both variables are on the scale of proportions, these values are comparable. We considered the composite median observed effect of LowSec+ Change as the median observed value of LowSec+ Change multiplied by the coefficient estimate for LowSec+ Change. Using the coefficient estimates from the main effects model, we found that the composite median observed effect of LowSec+ Change was 0.045×1.52=0.068. Analogously, we found the composite median observed effect of CP (Modern) Change was 0.0341×2.67=0.091. Even with a smaller observed value, CP (Modern) Change still had a larger composite median effect than LowSec+ Change. The same trends of larger median observed values of LowSec+ Change but larger composite median observed effect of CP (Modern) Change were found within SSA and within non‐SSA.

We did not find Child Mortality Decr to be significant despite the well‐documented marginal relationship between child mortality and fertility. The sign of the coefficient estimate for Child Mortality Decr was also opposite to what we would expect. This was unsurprising once we looked at the marginal relationship between Child Mortality Decr and TFR decrement. Although the nondecrement versions of child mortality and TFR were highly correlated at 0.77, the decrement versions only had a correlation of 0.10. However, when we considered the main effects model without the expected TFR decrement term (model 14a in in Table 8), we found Child Mortality Decr was significant. We also found the correlation between Expected TFR Decr and Child Mortality Decr was 0.27. These findings suggest that past trends in fertility decline may account for the explanatory potential of child mortality decrement on TFR decrement, thus leading to an insignificant result in the model including Expected TFR Decr.

Like Child Mortality Decr, Urban Change was not significant in the main effects model but was significant in the model without the expected TFR decrement term, suggesting the explanatory potential of the change over time in urbanization on fertility decline may be accounted for by the term representing past trends in fertility decline. As we did not find Urban Change to be significant in the model with main effects only, we believe the significance of Urban Change in the model with interactions is not of practical significance.

The control variables GDP Growth and GDP Growth Change must be interpreted together, as they measure aspects of the same quantity and have a significant positive correlation of 0.53. Due to the wide range of trends in GDP Growth and GDP Growth Change possible when a country is undergoing modernization, there is not a simple interpretation of the coefficients on GDP Growth and GDP Growth Change. The overall contribution of the GDP control variables to the predicted TFR decrement reflects both the growth rate and acceleration of GDP.

All of the interaction terms with the SSA indicator in the model with interactions implied a weaker relationship of the covariate on TFR decrement in SSA compared to non‐SSA. This will be explored further in a later section.

### Direct and indirect effects

We used path analysis to explore the structure of direct and indirect effects of our selected covariates on TFR decrement. Path analysis was developed by Wright (1921) as a way to decompose correlations between dependent and independent variables into direct and indirect effects. The results of a path analysis can be illustrated in a path diagram where unidirectional arrows are used to indicate the assumed causal relationships between covariates and bidirectional arrows are used to connect variables where there is no assumed causal relationship. Unidirectional arrows are labeled with standardized path coefficients, which are regression coefficients from regressions using standardized versions of the covariates, while bidirectional arrows are labeled with correlations.

A path diagram can illustrate the logical temporal ordering of the assumed causal pathway underlying our analyses. This ordering was arranged in levels in terms of proximity to TFR decrement, where Level 4 is the most proximate to fertility decline. No ordering is assumed among variables at the same level. The ordering was as follows:

Level 1: Urban Change, GDP Growth, GDP Growth Change.
Level 2: Education: LowSec+ Change.
Level 3: Child Mortality Decr.
Level 4: Family planning: CP (Modern) Change.

We included the three control variables measuring a form of “modernization” together on the same level. Among these modernization variables, we assumed a causal relationship only among covariates for Urban Change in the path diagram. Despite modernization being a central part of demographic transition theory, there is uncertainty about the direct effects of modernization variables on fertility decline (Hirschman 1994). There is greater support for an effect of urbanization on fertility decline in the literature (Garenne 2008; White et al. 2008; Bricker and Ibbitson 2019) than for measures of GDP. Thus, we did not make any assumption about the causal pathway between GDP and fertility decline. However, we still included the GDP variables as covariates in the regression for TFR decrement to ensure that the regression represented in the path diagram corresponded to the main effects model in Table 4. In the path diagram, we drew unidirectional arrows from Urban Change pointing towards covariates that were assumed to be more proximate to fertility decline. Connections between the GDP variables and all other covariates were assumed to be bidirectional arrows.

**FIGURE 5:**
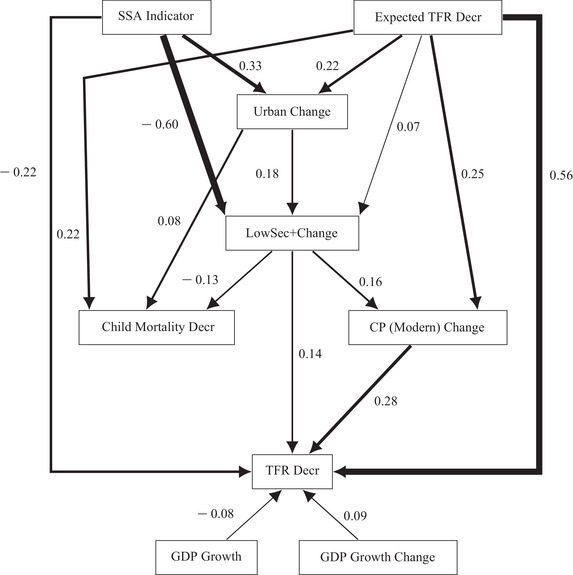
Path diagram with standardized path coefficients fit using GLS with error terms, bidirectional arrows, and arrows corresponding to effects with P>0.05 omitted for readability and line thicknesses proportional to path coefficient magnitudes

The path diagram is shown in Figure 5. All regressions in the path diagram include the SSA indicator and Expected TFR Decr as covariates, where the unidirectional arrows from SSA and Expected TFR Decr to all other variables in the path diagram represent assumptions on temporal ordering rather than causality. The direct effects of the GDP variables on TFR Decr are displayed in Figure 5, but the bidirectional arrows connecting GDP Growth and GDP Growth Change to SSA, Expected TFR Decr, Urban Change, LowSec+ Change, Child Mortality Decr, and CP (Modern) Change are omitted for readability. Error terms for Urban Change, LowSec+ Change, Child Mortality Decr, CP (Modern) Change, and TFR Decr are omitted from Figure 5 for readability. Arrows corresponding to effects with *P* >0.05 are also omitted from Figure 5 for readability. Path coefficients for the omitted bidirectional arrows, error terms, and arrows corresponding to effects with *P* >0.05 are reported in the online supplementary Appendix. The path coefficients displayed in Figure 5 are the standardized regression coefficients for models fit using GLS with the UN region × time point clustering scheme.

Figure 5 provides a visual representation of the relative strengths of the direct effects in the main effects only model summarized in Table 4 and the indirect effects between the covariates. We found that Expected TFR Decr had the largest direct effect on TFR decrement, as expected. The second largest direct effect on TFR decrement corresponded to contraceptive prevalence as measured by CP (Modern) Change. This effect was about twice as large as the direct effect of women's education as measured by LowSec+ Change.

In a traditional path diagram where path coefficients are obtained using ordinary least squares (OLS), the indirect effect of variable *X* on variable *Y* can be computed by multiplying the standardized path coefficients along the indirect path from *X* to *Y*. We used GLS with the UN region × time point clustering scheme to obtain our standardized path coefficients, which resulted in slightly different coefficient estimates from the OLS estimates. However, we can still estimate the indirect effects by multiplying path coefficients from GLS regressions. Comparing direct and indirect effects of women's education is of particular interest. We found that the direct effect of LowSec+ Change on TFR decrement was 0.14. The estimated indirect effect of LowSec+ Change through CP (Modern) Change was 0.16×0.28=0.0448, and the estimated indirect effect of LowSec+ Change through Child Mortality Decr was −0.13×−0.01=0.0013. Thus the direct effect of LowSec+ Change on TFR decrement is three times larger than its total indirect effect. Also, the indirect effect of LowSec+ Change is predominantly through CP (Modern) Change. This finding is in line with the literature, which suggests that one venue through which increased women's education impacts fertility decline is through increasing knowledge and acceptance of family planning. We also found that the direct effect of contraceptive prevalence (0.28) was greater than the sum of the direct and indirect effects of education (0.1861).

### SSA difference

We explore the effect of education and family planning on fertility decline within SSA by rewriting the model with SSA interactions in terms of one model for countries in SSA and one model for the rest of the world, displayed in Table 5.

**TABLE 5:** Comparison of coefficient estimates from the model with interactions in Table 4 for countries not in SSA and countries in SSA

	non‐SSA	SSA
(Intercept)	0.31	0.25
Expected TFR Decr	0.81	0.81
LowSec+ Change	1.79	1.22
CP (Modern) Change	3.38	1.83
GDP Growth	−1.20	−0.11
GDP Growth Change	0.77	0.16
Urban Change	−1.46	0.35
Child Mortality Decr	0.44	−0.96

We found that the effects of LowSec+ Change and CP (Modern) Change on TFR decrement were weaker in SSA than in non‐SSA countries, with a bigger decrease in effect size for CP (Modern) Change. According to our model, a faster increase in the proportion of women who have attained at least lower secondary education in SSA corresponds to a smaller decrease in TFR than what an increase in educational attainment of the same rate would have corresponded to in non‐SSA. Similarly, a faster increase in contraceptive prevalence in SSA corresponds to a smaller decrease in TFR than what an increase in contraceptive prevalence of the same rate would have corresponded to in non‐SSA. We also found a weaker effect of GDP Growth, GDP Growth Change, and Urban Change in SSA compared to countries not in SSA. The direction of the effect changed signs between SSA and non‐SSA for both Urban Change and Child Mortality Decr. The magnitude of the effect of Child Mortality Decr was larger in SSA than in non‐SSA; however from Table 4 we found that neither the main effect for Child Mortality Decr nor its interaction with SSA was significant.

The smaller effect size of LowSec+ Change on TFR Decr in SSA indicates the accelerating effect that increased women's attainment has on fertility decline is diminished in SSA compared to other high‐fertility regions. Martin (1995) also found the expected negative relationship between women's education and fertility to be weaker than anticipated in SSA compared to trends from historical high‐fertility transitions in non‐SSA regions. The weaker effect of women's education on fertility decline in SSA may be due to reductions in school quality or limited expansion of the labor market in SSA (Grant 2015). These same factors that affect the strength of the association between women's education and fertility may result in a weaker accelerating effect of education as well.

The weaker effect of CP (Modern) Change we found for SSA compared to non‐SSA refers only to the accelerating effect that increased contraceptive prevalence may have on fertility. Several studies on the effect of contraceptive prevalence on TFR in SSA have found a weaker effect than expected compared to global trends (Bongaarts 1987; Tsui 2001; Westoff and Bankole 2001). Bongaarts (2017) outlined technical and methodological pitfalls that may bias analyses of the relationship between contraceptive prevalence and TFR in SSA, arguing that the average effect of contraceptive prevalence on TFR is actually the same between SSA and non‐SSA after making data adjustments and controlling for regional fixed effects.

The major methodological pitfall discussed in Bongaarts (2017) is the confounding of cross‐sectional effect estimates with between‐country fertility differences that are constant over time. This is avoided in our analysis since we estimate the relationship between the change over time of contraceptive prevalence and the change over time of TFR rather than the relationship between contraceptive prevalence and TFR. The potential confounding bias is further reduced through the incorporation of country‐specific effects in our model via the country‐specific expected TFR decrement term.

Our work still has some of the technical limitations discussed by Bongaarts (2017). For TFR, we alleviate data quality issues and the delayed impact of contraception on TFR estimates by using estimates of TFR for five‐year periods from the UN. However, we were unable to implement all of Bongaarts’ suggestions for adjusting estimates of contraceptive prevalence. We aggregated yearly estimates of contraceptive prevalence of modern methods into five‐year periods to enable direct comparisons with the TFR for five‐year periods. The overlap with postpartum infecundability is partially addressed by the use of five‐year periods and the differences in contraceptive effectiveness of different contraceptive methods are partially addressed by focusing on modern methods only, as the least effective contraceptive methods are all classified as traditional methods by the UN.

Our chosen measure of contraceptive prevalence is limited by an incomplete exposure of risk to pregnancy and may experience confounding effects from variability in the age structures of women of reproductive age. While estimates of contraceptive prevalence for all women (whether or not they are married or in‐union) are available from the UN, these estimates are only available starting from 1990. When we used contraceptive prevalence estimates for all women to fit the model with SSA interactions, we still found a significant difference in the effect of CP (Modern) Change in SSA compared to non‐SSA, with a smaller effect size in SSA. Thus we chose to use estimates of contraceptive prevalence for married or in‐union women to make use of the additional data covering years 1970–1990. Details of the model using contraceptive prevalence estimates for all women can be found in the online supplementary Appendix. Also, the estimates of contraceptive prevalence are affected by the age distribution of women within each country while the estimates of TFR are not. This difference may result in a confounding effect of age structure on estimates of the relationship between CP (Modern) Change and TFR Decr.

The direct comparison in Table 5 is illuminating, but does not explain the difference in the average rate of fertility decline between non‐SSA and SSA. To explore potential explanations for this difference, we considered sequential models of TFR decrement that added covariates in one at a time. The order in which covariates were added was chosen to reflect the logical temporal ordering of the potentially causal relationships between the covariates and TFR decrement. We used the same order as was used for the path analysis. Note that the three control variables measuring “modernization” (Urban Change, GDP Growth, and GDP Growth Change) do not have an intrinsic temporal ordering. As we added covariates one at a time into the sequential models, we made an arbitrary selection of the order in which to add the three modernization control variables. We chose the following ordering: SSA indicator, Urban Change, GDP Growth, GDP Growth Change, LowSec+ Change, Child Mortality Decr, and finally CP (Modern) Change. After comparing the main effects, we also considered all interactions with the SSA indicator. These interactions were added in the same temporal order. All sequential models were fit using GLS with the UN region × time point clustering scheme, and all continuous variables were centered prior to fitting the models.

We considered these sequential models both with and without the expected TFR decrement term. Models without the expected TFR decrement term can show general trends without taking historical TFR trajectories into account and provide a descriptive account of relationships between the covariates and TFR decrement. The results of the sequential models without the expected TFR decrement term can be directly compared to existing work investigating how the SSA fertility decline differs from other historical fertility declines, such as Bongaarts and Casterline (2013). The sequential models with Expected TFR Decr are summarized in Tables 6 and 7. The sequential models without Expected TFR Decr are summarized in Tables 8 and 9.

**TABLE 6:** Comparison of sequential models with Expected TFR Decr and with main effects only, fit via GLS with TFR decrement as the dependent variable

Sequential models	Model 1	Model 2a	Model 3a	Model 4a	Model 5a	Model 6a	Model 7a
(Intercept)	0.33^***^	0.33^***^	0.33^***^	0.33^***^	0.31^***^	0.30^***^	0.31^***^
Expected TFR Decr	0.94^***^	0.94^***^	0.94^***^	0.94^***^	0.93^***^	0.92^***^	0.82^***^
SSA	−0.09^**^	−0.09^**^	−0.10^**^	−0.10^**^	−0.07^*^	−0.07^*^	−0.07^*^
Urban Change		−0.01	0.01	0.20	−0.26	−0.29	−0.35
GDP Growth			−0.06	−0.34	−0.36	−0.40	−0.58^*^
GDP Growth Change				0.38^*^	0.43^*^	0.44^*^	0.51^**^
LowSec+ Change					1.98^***^	2.03^***^	1.52^***^
Child Mortality Decr						0.81	−0.14
CP (Modern) Change							2.67^***^
*R* ^2^	0.45	0.45	0.45	0.45	0.48	0.48	0.55

^***^denotes P<0.001, ^**^denotes P<0.01, and ^*^denotes P<0.05.

**TABLE 7:** Comparison of sequential models with Expected TFR Decr and with interactions, fit via GLS with TFR decrement as the dependent variable

Sequential Models	Model 1	Model 2b	Model 3b	Model 4b	Model 5b	Model 6b	Model 7b
(Intercept)	0.33^***^	0.32^***^	0.32^***^	0.32^***^	0.30^***^	0.30^***^	0.31^***^
Expected TFR Decr	0.94^***^	0.95^***^	0.95^***^	0.94^***^	0.93^***^	0.89^***^	0.81^***^
SSA	−0.09^**^	−0.10^**^	−0.10^**^	−0.10^**^	−0.07^*^	−0.06	−0.06^*^
Urban Change		−0.45	−0.46	−0.24	−0.86	−1.09	−1.46^*^
GDP Growth			−0.31	−0.97^*^	−0.91^*^	−1.02^**^	−1.20^**^
GDP Growth Change				0.68^**^	0.68^**^	0.73^**^	0.77^***^
LowSec+ Change					2.02^***^	2.09^***^	1.79^***^
Child Mortality Decr						3.01^*^	0.44
CP (Modern) Change							3.38^***^
SSA:Urban Change		0.95	0.91	0.67	1.06	1.26	1.81^*^
SSA:GDP Growth			0.39	1.07^*^	0.90	1.07^*^	1.08^*^
SSA:GDP Growth Change				−0.71	−0.56	−0.65	−0.61
SSA:LowSec+ Change					−0.15	−0.24	−0.57
SSA:Child Mortality Decr						−3.59^*^	−1.40
SSA:CP (Modern) Change							−1.55^*^
*R* ^2^	0.45	0.45	0.45	0.46	0.49	0.49	0.57

^***^denotes P<0.001, ^**^denotes P<0.01, and ^*^denotes P<0.05.

**TABLE 8:** Comparison of sequential models without Expected TFR Decr and with main effects only, fit via GLS with TFR decrement as the dependent variable

Sequential Models	Model 8	Model 9a	Model 10a	Model 11a	Model 12a	Model 13a	Model 14a
(Intercept)	0.42^***^	0.42^***^	0.42^***^	0.42^***^	0.39^***^	0.38^***^	0.38^***^
SSA	−0.11^*^	−0.12^**^	−0.12^**^	−0.13^**^	−0.09^*^	−0.10^*^	−0.09^**^
Urban Change		2.00^***^	2.14^***^	2.34^***^	1.72^**^	1.44^*^	1.10^*^
GDP Growth			−0.52	−0.82^*^	−0.82^*^	−1.03^**^	−1.21^***^
GDP Growth Change				0.40	0.46	0.53^*^	0.61^**^
LowSec+ Change					2.45^***^	2.71^***^	1.87^***^
Child Mortality Decr						4.94^***^	3.03^**^
CP (Modern) Change							3.87^***^
*R* ^2^	0.04	0.05	0.06	0.06	0.12	0.15	0.29

^***^denotes P<0.001, ^**^denotes P<0.01, and ^*^denotes P<0.05.

**TABLE 9:** Comparison of sequential models without Expected TFR Decr and with interactions, fit via GLS with TFR decrement as the dependent variable

Sequential Models	Model 8	Model 9b	Model 10b	Model 11b	Model 12b	Model 13b	Model 14b
(Intercept)	0.42^***^	0.42^***^	0.42^***^	0.42^***^	0.40^***^	0.37^***^	0.37^***^
SSA	−0.11^*^	−0.11^*^	−0.11^*^	−0.10^*^	−0.07	−0.04	−0.05
Urban Change		2.85^***^	2.77^***^	3.02^***^	2.39^**^	0.99	0.30
GDP Growth			−1.08^*^	−1.97^***^	−1.89^***^	−2.15^***^	−2.22^***^
GDP Growth Change				0.92^**^	0.92^**^	1.10^***^	1.08^***^
LowSec+ Change					2.01^***^	2.30^***^	1.86^***^
Child Mortality Decr						11.44^***^	7.30^***^
CP (Modern) Change							4.29^***^
SSA:Urban Change		−1.92	−1.92	−2.54^*^	−2.25	−0.90	0.05
SSA:GDP Growth			1.21^*^	2.43^***^	2.17^**^	2.46^***^	2.26^***^
SSA:GDP Growth Change				−1.45^**^	−1.21^*^	−1.38^**^	−1.23^**^
SSA:LowSec+ Change					0.85	0.51	−0.09
SSA:Child Mortality Decr						−11.99^***^	−8.22^***^
SSA:CP (Modern) Change							−1.59^*^
*R* ^2^	0.04	0.06	0.07	0.08	0.13	0.21	0.33

^***^denotes P<0.001, ^**^denotes P<0.01, and ^*^denotes P<0.05.

**FIGURE 6:**
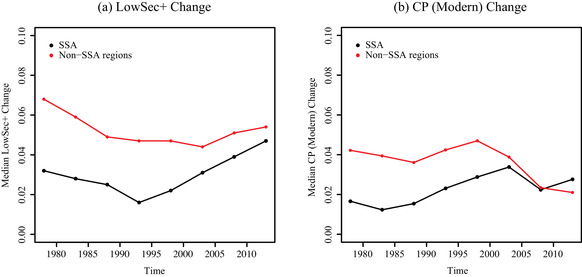
Comparison of median trends in LowSec+ Change and CP (Modern) Change for SSA (black) and non‐SSA (red) from 1975–1980 to 2010–2015

For all the sequential models, the biggest change in the SSA coefficient estimate came from adding LowSec+ Change into the model. Thus we found that the difference in the average rate of fertility decline between SSA and non‐SSA could be partially explained by differences in trends in LowSec+ Change between the two geographic areas. This is illustrated in Figure 6a, where the median trends in LowSec+ Change for SSA and non‐SSA are plotted over time. Although there are clearly differences in the median trends in LowSec+ Change, with larger increases in attainment in non‐SSA, the difference appears to be narrowing over time.

We did not find much change in the SSA coefficient estimate when we added in CP (Modern) Change, although we did see an increase in *R* ^2^ in all models. In Figure 6b, we see that the median trends in CP (Modern) Change for SSA and non‐SSA have some overlap. However, we did find a significant difference in the way changes to CP (Modern) Change impact TFR Decrement in SSA compared to non‐SSA when we considered the sequential models with interactions both with and without the expected TFR decrement. We found that increases in CP (Modern) have a smaller effect on TFR Decrement in SSA when compared to non‐SSA. This is in line with the findings of Bongaarts and Casterline (2013), which point to the high ideal family size in Africa as an obstacle to accelerated fertility decline even in the presence of low unmet need for contraception.

We found similar results for the models with and without Expected TFR Decr, where the only major differences centered around Urban Change and Child Mortality Decr. Coefficient estimates for LowSec+ Change and CP (Modern) Change were consistent across all models with significant positive effects and a larger effect size for CP (Modern) Change compared to LowSec+ Change. The effect sizes for both LowSec+ Change and CP (Modern) Change were smaller in the models with Expected TFR Decr compared to the models without Expected TFR Decr, indicating that some of the positive association of increased educational attainment and increased contraceptive prevalence with fertility decline can be accounted for by past trends in fertility decline. However, we still observed significant, positive effects for LowSec+ Change and CP (Modern) Change in the models including Expected TFR Decr and observed similar relative effect sizes of LowSec+ Change and CP (Modern) Change in the models with and without Expected TFR Decr. Similarly, both GDP Growth and GDP Growth Change were significant in the models with and without Expected TFR Decr; however, the effect sizes were smaller in magnitude in the models with Expected TFR Decr.

The differences in the coefficient estimates for Child Mortality Decr and Urban Change between the models with and without the expected TFR decrement were larger. In the sequential models without the expected TFR decrement term, we found a significant effect of Child Mortality Decr and coefficient estimates that reflected the expected positive relationship between Child Mortality Decr and TFR decrement. However, once Expected TFR Decr was included in the models, the child mortality term was no longer significant and in some cases had the wrong sign on the coefficient estimates. We observed similar differences for Urban Change. While the models including the expected TFR decrement term allow us to examine the additional impact covariates may have on fertility decline beyond what we would already expect the decline to look like based on past trends, the models without the expected TFR decrement term only describe the overall associations between fertility decline and the covariates. Thus, the differences in significance for Child Mortality Decr and Urban Change may indicate that once we account for past trends in fertility decline via the expected TFR decrement term, Child Mortality Decr and Urban Change do not provide any additional information about changes in TFR in a Granger causality context.

## Discussion

Our analyses aimed to estimate the effects of education and family planning on fertility decline in a high‐fertility context, with a focus on the accelerating effect of education and family planning on TFR decline. For education, we aimed to determine whether the effect of education operates through increased educational attainment of women or through increased educational enrollment of children. We were also interested in determining which educational level has the strongest impact on fertility decline. For family planning, we aimed to assess whether the effect of family planning operates by reducing unmet need or by increasing the prevalence of modern contraceptive methods. We also aimed to compare the effects of education and family planning to determine which contributes more to accelerating fertility decline.

We found significant accelerating effects of educational attainment and contraceptive prevalence. Specifically, we found that larger rates of increase in the proportion of women who have attained lower secondary education or higher corresponded to faster declines in TFR. We found a separate accelerating effect of increasing contraceptive prevalence of modern contraceptive methods. Contraceptive prevalence had the largest effect size of all covariates we considered, including education. We found that the effect size for contraceptive prevalence was larger than the effect size for education even when taking the smaller observed values for contraceptive prevalence compared to the observed values for women's attainment into account. Using path analysis, we found a small indirect effect of women's educational attainment on fertility decline through contraceptive prevalence, but the direct effect of education was three times larger than the total indirect effect of education.

The accelerating effects we found were dampened within SSA compared to the rest of the world. This dampening is partly explained by differences in the pace of change of women's educational attainment between SSA and non‐SSA countries. However, the amount of the average difference in TFR decrement that can be explained by the educational differences is small, and the differences in trends in the rate of change of women's attainment between SSA and non‐SSA are narrowing over time.

Our approach is inspired by Granger causality and so it attempts to estimate causal effects. However, this does not fully exclude the possibility of the results being affected by unobserved confounders, although the risk of this is smaller than with a traditional regression analysis. Thus, caution is needed when interpreting the estimated parameters.

Nevertheless, our findings do suggest several possible implications for policies aimed at accelerating fertility decline. First, it is women's attainment, not children's enrollment, that leads to accelerated rates of fertility decline. Of the different education levels, we found that lower secondary education had the most important accelerating effect. Primary education had a much smaller effect, and additional education beyond the lower secondary level (typically around ages 14–16) also had a smaller effect. Lower secondary education is generally considered the final stage of basic education, and this suggests that making completion of lower secondary education universal throughout the world would accelerate fertility decline. This is Target 4.1 of the Sustainable Development Goals.

We found that women's education and contraceptive prevalence both had significant effects, with contraceptive prevalence having a substantially larger effect size. Finally, policies leading to increases in education and family planning within currently high‐fertility countries in SSA may have a lessened effect on fertility decline than has previously been seen from similar policies in other historically high‐fertility regions. For education, this may partly reflect differences in educational quality (Grant 2015), suggesting a focus on improving educational quality in SSA.

## Supporting information

Supporting Information
