# Supplementary material for: How Do Education and Family Planning Accelerate Fertility Decline?
Source: Popul Dev Rev. 2020 Jul 23;46(3):409–41. doi: 10.1111/padr.12347 (PMC7590131; doi:10.1111/padr.12347)
Supplement: Supplementary file 1 — Supporting Information [file PADR-46-409-s001.pdf]

Appendix A: Verification of Model Selection for Education Using Non-cumulative Levels of Attainment

In the model selection for education, we only considered the cumulative levels of change over time in women's educational attainment for interpretation purposes. However, we note that the cumulative levels of attainment are subsets of one another. For example, LowSec+ Change is the change over time in the proportion of women who have attained lower secondary education or higher. Both UppSec+ Change, the change over time in the proportion of women who have attained upper secondary education or higher, and PostSec+ Change, the change over time in the proportion of women who have attained post secondary education, are subsets of LowSec+ Change. Although we selected from the cumulative parameterization of the change over time in women's attainment ("Attain+ Change"), we also looked at the estimated effects for the non-cumulative parameterization ("Attain Change") as to verify our variable selection results.

Table T1 summarizes the model including variables measuring women's attainment and children's enrollment. For comparison purposes, Table T1 contains both the model that uses Attain+ Change and the model that uses Attain Change. The different levels of women's attainment are abbreviated analogously to "LowSec" for lower secondary. The change over time in Net Enrollment Rate is abbreviated to NER Change.

In both the Attain+ Change and Attain Change versions of the model in Table T1, we found the only significant education variables corresponded to the change over time in women's attainment for lower secondary education or higher. In the cumulative attainment model, this was LowSec+ Change. In the non-cumulative attainment model, this was LowSec Change, UppSec Change, and PostSec Change. Neither of the variables measuring children's enrollment were significant. Since we constructed the "change over time" education variables to be positive when education is increasing, we expected to find positive coefficient estimates for the education variables. However, we found that several of the coefficient estimates, including the coefficient estimates for both enrollment variables, were negative.

From the results in Table T1, we have verified that women's attainment was selected over children's enrollment regardless of the parameterization of women's attainment. We also found the only significant levels of attainment corresponded to the levels lower secondary or higher for both parameterizations of women's attainment. However, due to the limited availability of data on children's enrollment, the models in Table T1 were fit using only 550 country-time pairs. Given that the selected education mechanism was attainment, we confirmed that the selected levels of attainment were truly lower secondary or higher once we considered all 666 country-time pairs. Table T2 summarizes the model not including

Table T1: Summary of model fit with all education variables and all control variables, fit by GLS with TFR decrement as the dependent variable

Model with Attain+ Change and NER Change	Estimate	t-value	Model with Attain Change and NER Change	Estimate	t-value
(Intercept)	0.33	17.4***	(Intercept)	0.33	17.4***
Expected TFR Decr	0.91	18.4***	Expected TFR Decr	0.91	18.4***
IncPri+ Change	−0.66	−1.3	IncPri Change	−0.66	−1.3
Pri+ Change	0.79	1.3	Pri Change	0.13	0.3
LowSec+ Change	2.11	3.2**	LowSec Change	2.25	3.7**
UppSec+ Change	−0.78	−1.0	UppSec Change	1.47	2.1*
PostSec+ Change	0.63	0.5	PostSec Change	2.10	2.4*
NER Change (Pri)	−0.16	−1.1	NER Change (Pri)	−0.16	−1.1
NER Change (Sec)	−0.16	−1.6	NER Change (Sec)	−0.16	−1.6
GDP Growth	−0.38	−1.4	GDP Growth	−0.38	−1.4
GDP Growth Change	0.55	2.9**	GDP Growth Change	0.55	2.9**
Urban Change	−0.31	−0.6	Urban Change	−0.31	−0.6
Child Mortality Decr	0.07	0.1	Child Mortality Decr	0.07	0.1
SSA	−0.08	−2.5*	SSA	−0.08	−2.5*
Within-cluster Correlation	0.23		Within-cluster Correlation	0.23	
R^2	0.52		R^2	0.52	
BIC	-52.30		BIC	-52.30	
Country-time pairs	550		Country-time pairs	550	

*** denotes P< 0.001, ** denotes P< 0.01, and * denotes P< 0.05

enrollment variables. We considered versions with Attain+ Change and Attain Change for comparison purposes. In the model with Attain+ Change, we found LowSec+ Change was the only significant education variable. In the model with Attain Change, we found the education level with the largest effect size was lower secondary. The next largest effect sizes corresponded to upper secondary and post secondary. Although PostSec Change was not significant in the model with Attain Change, we justified the choice of LowSec+ Change since the women who have attained post secondary education have necessarily also attained lower secondary and upper secondary education, both of which were significant. The results of the comparison in Table T2 support our choice of LowSec+ Change as the selected education variable and confirm that lower secondary is the most important level of education in terms of effect size.

Table T2: Summary of model fit with attainment variables and all control variables, fit by GLS with TFR decrement as the dependent variable

Model with Attain+ Change			Model with Attain Change		
	Estimate	t-value		Estimate	t-value
(Intercept)	0.30	16.3***	(Intercept)	0.30	16.3***
Expected TFR Decr	0.91	19.2***	Expected TFR Decr	0.91	19.2***
IncPri+ Change	−0.73	−1.5	IncPri Change	−0.73	−1.5
Pri+ Change	1.00	1.8	Pri Change	0.27	0.7
LowSec+ Change	2.38	3.9***	LowSec Change	2.65	4.8***
UppSec+ Change	−1.04	−1.5	UppSec Change	1.62	2.6**
PostSec+ Change	−0.49	−0.5	PostSec Change	1.13	1.4
GDP Growth	−0.45	−1.7*	GDP Growth	−0.45	−1.7*
GDP Growth Change	0.46	2.6*	GDP Growth Change	0.46	2.6*
Urban Change	−0.47	−1.0	Urban Change	−0.47	−1.0
Child Mortality Decr	0.70	0.9	Child Mortality Decr	0.70	0.9
SSA	−0.09	−2.6**	SSA	−0.09	−2.6**
Within-cluster Correlation	0.30		Within-cluster Correlation	0.30	
R^2	0.49		R^2	0.49	
BIC	-86.45		BIC	-86.45	
Country-time pairs	666		Country-time pairs	666	

*** denotes P< 0.001, ** denotes P< 0.01, and * denotes P< 0.05

Appendix B: Contraceptive Prevalence for All Women

In our analyses, we used estimates of contraceptive prevalence from the UN for married or in-union women aged 15-49 years, available from 1970 onwards. A technical limitation of using these estimates is an incomplete exposure to risk of pregnancy, as the TFR measures births to all women and not only women who are married or in-union (Bongaarts 2017). This limitation may have a downward bias on the estimated effect of contraceptive prevalence on TFR within SSA compared to the rest of the world (Bongaarts 2017). The UN also provides estimates of contraceptive prevalence for all women (whether or not they are married or in-union), however these estimates are only available from 1990 onwards. This restriction reduces the size of our dataset substantially. When using the married or in-union estimates, we have 666 country-time pairs with observations from 121 countries. However, when using the estimates for all women, we only have 344 country-time pairs with observations from 104 countries.

To check if using contraceptive prevalence for married or in-union women has a downward bias on the estimated effect of CP (Modern) Change on TFR Decr within SSA compared to non-SSA, we compared the model with CP (Modern) Change for married or in-union women with the analogous model fit using CP (Modern) Change for all women. The model was fit using GLS with the UN Region × Time Point clustering scheme and includes LowSec+ Change, the SSA indicator, Expected TFR Decr, GDP Growth, GDP Growth Change, Urban Change, and Child Mortality Decr as additional covariates. All continuous variables were centered prior to model fitting. Table T3 provides a comparison of the results of the model using contraceptive prevalence for married or in-union women with the equivalent model fit using contraceptive prevalence for all women.

When considering the contraceptive prevalence of married or in-union women, the estimated effect of CP (Modern) Change on TFR Decr was 1.83 in SSA and 3.38 in non-SSA, which is an estimated effect about 1.85 times larger in non-SSA than in SSA. When considering the contraceptive prevalence of all women, the estimated effect of CP (Modern) Change on TFR Decr was 2.29 in SSA and 4.06 in non-SSA, which is an estimated effect about 1.77 times larger in non-SSA than in SSA. We found a notable and significant difference in the effect of CP (Modern) Change on TFR Decr between SSA and non-SSA regardless of which measure of contraceptive prevalence we considered. The difference in relative effect sizes between the two regions was smaller when we consider the contraceptive prevalence of all women, however the results still indicate the accelerating effect of increased contraceptive prevalence on fertility decline was weaker in SSA than in non-SSA. As the resulting conclusions about whether SSA is different remain the same regardless of which measure of

contraceptive prevalence was used, we chose to use contraceptive prevalence of married or in-union women to make use of data from 1970-1990.

Table T3: Comparison of models using contraceptive prevalence for married or in-union women and contraceptive prevalence for all women, fit by GLS with TFR decrement as the dependent variable

	Married or In-Union Women		All Women	
	Estimate	t-value	Estimate	t-value
(Intercept)	0.31	19.6***	0.32	15.0***
Expected TFR Decr	0.81	18.3***	1.11	15.6***
LowSec+ Change	1.79	4.6***	2.22	4.6***
CP (Modern) Change	3.38	9.3***	4.06	5.9***
GDP Growth	−1.20	−3.3**	−1.50	−2.7**
GDP Growth Change	0.77	3.7***	0.94	3.3**
Urban Change	−1.46	−2.5*	−1.19	−1.5
Child Mortality Decr	0.44	0.4	2.93	1.4
SSA	−0.06	−2.1*	−0.11	−3.1
SSA:LowSec+ Change	−0.57	−0.8	−1.25	−1.7
SSA:CP (Modern) Change	−1.55	−2.8**	−1.76	−2.0*
SSA:GDP Growth	1.08	2.1*	1.48	2.3*
SSA:GDP Growth Change	−0.61	−1.7	−0.94	−2.2*
SSA:Urban Change	1.81	2.1*	2.15	1.7
SSA:Child Mortality Decr	−1.40	−0.9	−3.65	−1.5
Within-cluster Correlation	0.23		0.31	
R^2	0.57		0.61	
BIC	-168.49		-147.40	

*** denotes P< 0.001, ** denotes P< 0.01, and * denotes P< 0.05

References

Bongaarts, John. 2017. "The Effect of Contraception on Fertility: Is Sub-Saharan Africa Different?" *Demographic Research* 37 (6): 129–146. https://doi.org/10.4054/DemRes.2017.37.6.

Appendix C: Omitted Path Coefficients

The bidirectional arrows connecting GDP Growth and GDP Growth Change to SSA, Expected TFR Decr, Urban Change, LowSec+ Change, Child Mortality Decr, and CP (Modern) Change are omitted from the path diagram for readability. The path coefficients for the omitted bidirectional arrows correspond to the correlations reported in Table T4.

Error terms for Urban Change, LowSec+ Change, Child Mortality Decr, CP (Modern) Change, and TFR Decr are omitted for readability. The path coefficients for the omitted error terms are reported in Table T5.

Arrows corresponding to effects with $P > 0.05$ are also omitted for readability. The path coefficients for the unidirectional arrows going from the "starting" variable to the "ending" variable are reported in Table T6

Table T4: Path coefficients for bidirectional arrows (omitted from path diagram for readability) from GDP Growth and GDP Growth Change to all other covariates

	GDP Growth Coefficient	GDP Growth Change Coefficient
SSA	-0.03	0.02
Expected TFR Decr	-0.06	-0.07
Urban Change	0.11	-0.09
LowSec+ Change	0.05	-0.08
Child Mortality Decr	0.11	-0.02
CP (Modern) Change	0.08	-0.01
GDP Growth	—	0.53
GDP Growth Change	0.53	—

Table T5: Path coefficients for error terms (omitted from path diagram for readability)

	Error Term Coefficient
Urban Change	0.97
LowSec+ Change	0.93
Child Mortality Decr	0.94
CP (Modern) Change	0.94
TFR Decr	0.67

Table T6: Path coefficients for unidirectional arrows from “starting” variable to “ending” variable corresponding to effects with P > 0.05 (omitted from path diagram for readability)

Starting Variable	Ending Variable	Path Coefficient
SSA Indicator	Child Mortality Decr	0.21
SSA Indicator	CP (Modern) Change	0.01
Urban Change	CP (Modern) Change	0.04
Urban Change	TFR Decr	-0.02
Child Mortality Decr	TFR Decr	-0.01
